# Distinct MAIT cell phenotypes associated with sepsis clinical outcome in emergency department patients

**DOI:** 10.1002/cti2.70028

**Published:** 2025-03-04

**Authors:** Johanna Emgård, Iva Filipovic, Christian Unge, Laura M Palma Medina, Åsa Parke, Helena Bergsten, Kirsten Moll, Majda Dzidic, Helena Alpkvist, Hong Fang, Volkan Özenci, Niklas K Björkström, Mattias Svensson, Johan K Sandberg, Kristoffer Strålin, Anna Norrby‐Teglund

**Affiliations:** ^1^ Department of Medicine Huddinge, Center for Infectious Medicine, Karolinska University Hospital Karolinska Institutet Stockholm Sweden; ^2^ Department of Medicine Huddinge Karolinska Institutet Stockholm Sweden; ^3^ Functional Area of Emergency Medicine Karolinska University Hospital Stockholm Sweden; ^4^ Department of Infectious Diseases Karolinska University Hospital Stockholm Sweden; ^5^ Division of Clinical Microbiology, Department of Laboratory Medicine Karolinska Institutet Stockholm Sweden; ^6^ Department of Clinical Microbiology Karolinska University Hospital Stockholm Sweden

**Keywords:** immunophenotyping, MAIT cells, sepsis, sepsis endotypes

## Abstract

**Objectives:**

Rapid diagnosis and intervention are critical for sepsis patient outcomes. However, diagnosis is challenging because of a heterogenic patient group as well as sometimes vague symptoms when the patient presents at the emergency department. Mucosal‐associated invariant T (MAIT) cells are rapid responders to infection, but their role and characteristics in the early course of sepsis remain unknown. Here, we evaluate the early MAIT cell characteristics in the blood of patients triggering a clinical sepsis alert system at the emergency department.

**Methods:**

Peripheral blood mononuclear cells were isolated from freshly drawn blood and immediately stained. MAIT cell phenotyping analyses were conducted using multiparameter flow cytometry. All analyses were completed prior to the stratification of patients into sepsis or non‐sepsis groups. Soluble factors in plasma were measured using a multiplex assay.

**Results:**

Unsupervised high‐dimensional phenotyping identified distinct MAIT cell activation profiles in sepsis and non‐sepsis groups. Among sepsis patients, hierarchical clustering of MAIT cell phenotypes separated clinical endotypes into three groups with different infection focus, severity and aetiology. A prominent characteristic of sepsis severity was high expression of CD69 on MAIT cells, which was associated with organ dysfunction, lymphopenia and poor outcome. Plasma levels of IL‐12, IL‐15, TNF, IFNγ and CXCL10 correlated with the magnitude of MAIT cell activation in sepsis patients.

**Conclusions:**

These clinical endotype‐specific MAIT cell phenotypes presenting already in the emergency department are of interest for early patient identification and prognostication in sepsis.

## Graphical abstract

In this study, mucosal‐associated invariant T (MAIT) cell characteristics were evaluated in blood from patients who triggered a clinical sepsis alert at the emergency department. High‐dimensional phenotyping revealed distinct MAIT cell activation profiles between sepsis and non‐sepsis groups. In sepsis patients, hierarchical clustering identified three clinical endotypes with different infection focuses, severity and aetiology, which are of interest for early patient identification and prognostication.

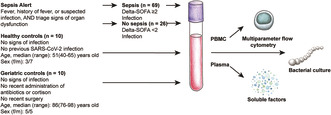

## Introduction

Sepsis is a life‐threatening condition defined as organ dysfunction caused by a dysregulated host response to an infection^1^ and a leading cause of death among hospitalised patients.^2^ Sepsis is a highly heterogeneous syndrome deriving from a wide range of bacterial, viral or fungal infections. Infections of the respiratory tract are the leading cause, followed by urinary tract infections and intra‐abdominal infections.^3^ The pathobiology is complex and still not fully defined. What was earlier believed to be primarily a hyperinflammatory condition is now recognised to involve both pro‐ and anti‐inflammatory responses, as well as alterations in metabolic, cardiovascular, hormonal, neuronal and coagulation pathways, and the state of the immune response changes over time.^1^, ^2^, ^3^, ^4^ On the one hand, the hyperinflammatory state is driven by massive immune cell activation and cytokine release, which might support pathogen eradication but at the same time damage tissues and result in organ dysfunction. On the other hand, the anti‐inflammatory response and immunosuppression make the patient more susceptible to secondary infections.^5^ As sepsis is a rapidly progressing condition, early identification and intervention in the first few hours are critical for patient survival.^6^ However, diagnosis may be challenging because of nonspecific initial symptoms and lack of sepsis‐specific diagnostic tools.^3^, ^7^

MAIT cells are rapid and early responders to both bacterial and viral infections.^8^, ^9^, ^10^ They are unconventional, innate‐like T cells that recognise microbial vitamin B metabolite‐derived antigens presented by the MHC class I‐related molecule MR1.^11^, ^12^ *Staphylococcus aureus*, *Streptococcus pneumoniae* and *Escherichia coli*, three of the most common causes of sepsis, all produce the antigens recognised by MAIT cells.^13^, ^14^ In addition, MAIT cells can respond to cytokines including IL‐12, IL‐18, IFN‐α and IL‐15,^10^, ^15^, ^16^, ^17^ all of which are elevated in the blood early in sepsis.^18^ Activated MAIT cells produce inflammatory cytokines including IFNγ, TNF and IL‐17A, and cytotoxic molecules such as granzyme B and perforin^14^, ^19^, ^20^ and are major contributors to the cytokine storm of both streptococcal and staphylococcal toxic shock syndrome (TSS).^21^, ^22^ Studies in sepsis patients admitted to the intensive care unit (ICU) have reported that MAIT cells are highly activated and decline in frequency in circulation.^23^, ^24^, ^25^, ^26^, ^27^, ^28^ However, the presence and phenotype of MAIT cells during early phases of sepsis, that is in the emergency department, are currently unknown. As MAIT cells are rapid responders to infection, they have the potential to serve as early biomarkers in this critical window for diagnosis and prognosis.

In this study, we therefore evaluate the potential of the MAIT cell phenotype in diagnosis and prognostication in patients with clinically suspected sepsis presenting at the emergency department. Early MAIT cell profiling allowed distinction between sepsis and non‐sepsis groups prior to clinical stratification. Among sepsis patients, MAIT cell phenotypes were associated with specific clinical endotypes.

## Results

### Demographic and clinical characteristics of study subjects

**Figure 1:**
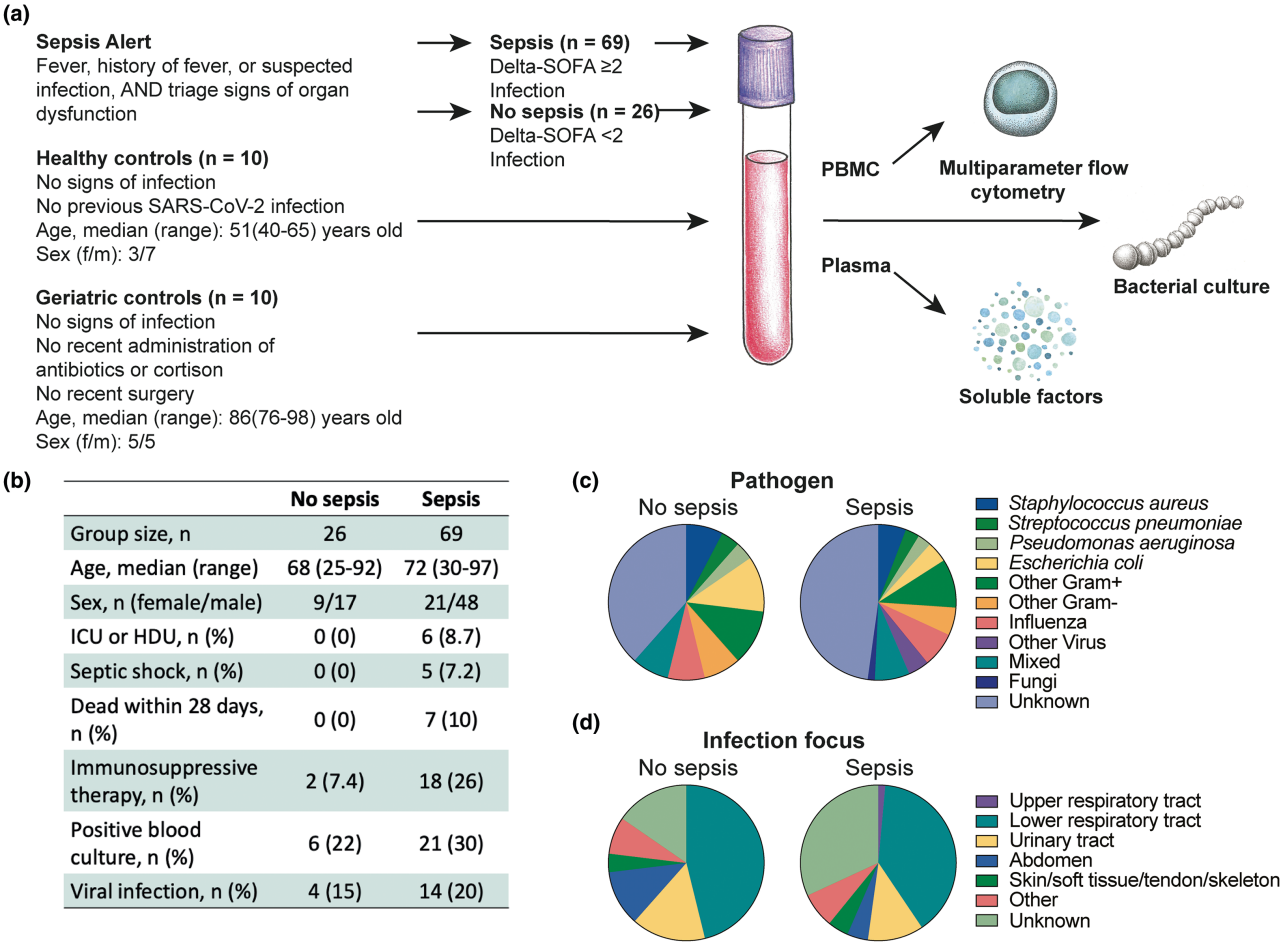
Study design and patient characteristics. **(a)** Schematic overview of study design, inclusion and exclusion criteria. **(b)** Patient characteristics and number of patients within the patient subgroups. **(c, d)** The distribution of **(c)** patients infected with indicated pathogens and **(d)** patients with indicated infection focuses within the sepsis and no sepsis groups.

To study the early immune profile of MAIT cells in sepsis, blood samples were collected at the emergency department from patients with clinically suspected sepsis recruited through the sepsis alert system (Supplementary table 1). The clinical stratification of patients into sepsis and non‐sepsis and into sepsis subgroups was completed after the samples had been analysed (Figure 1a). Characteristics of the patients are shown in Figure 1b, Supplementary table 2. Samples were also collected from two control groups: one with healthy donors below 65 years of age, collected from the blood bank, and one of patients above 65 years, without infection, collected from the geriatric ward (Figure 1a, Supplementary tables 1 and 2).

*Staphylococcus aureus* was the most common finding in blood cultures of sepsis patients, while *Escherichia coli* was more common in patients without sepsis (Figure 1c). The lower respiratory tract, followed by the urinary tract, was the most common infection foci for both sepsis and non‐sepsis patients (Figure 1d, Supplementary table 2).

### Early decline of MAIT cells in the circulation of sepsis patients

**Figure 2:**
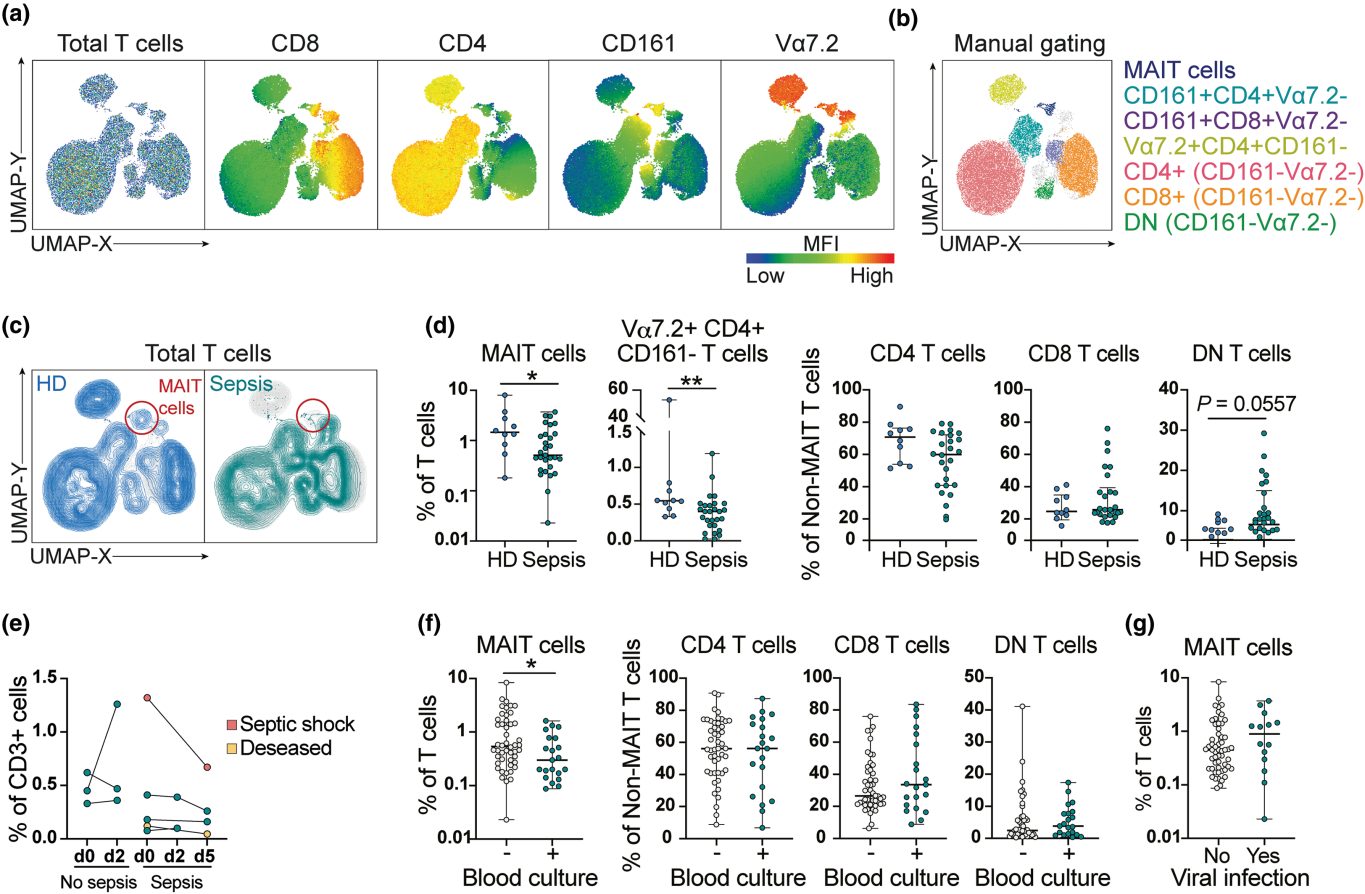
Decline of MAIT cell frequencies in sepsis patients compared to healthy donors. **(a)** UMAP plots of total live CD3^+^ cells in peripheral blood of healthy donors (HD) and sepsis patients below 65 years of age and expression of indicated markers. **(b)** Total CD3^+^ cells overlaid with immune cell subsets identified by manual gating. **(c)** Total CD3^+^ cells of healthy donors (blue) or sepsis patients below 65 years of age (green). Red circles indicate MAIT cells. **(d)** Frequencies of MAIT cells and Vα7.2^+^CD4^+^CD161^−^ cells among total CD3^+^ cells and frequencies of non‐MAIT CD4^+^, CD8^+^, and double negative (DN) T cells among non‐MAIT CD3^+^ cells of healthy donors and sepsis patients below 65 years of age, identified by manual gating. **(e)** Change in MAIT cell frequency among CD3^+^ cells over time in sepsis and no sepsis patients. Each line represents one patient. **(f)** Frequencies of MAIT cells among total CD3^+^ cells and frequencies of non‐MAIT CD4^+^, CD8^+^ and DN T cells among non‐MAIT CD3^+^ cells in sepsis patients with and without positive blood cultures. **(g)** MAIT cell frequency among CD3^+^ cells in patients with or without viral infection. Data in **(d)**, **(f)** and **(g)** are presented as median ± IQR and each dot represents an individual patient. Statistical analysis was performed using the nonparametric Mann–Whitney test. Statistically significant differences are indicated by **P < 0.01, *P < 0.05. In case of borderline significance, i.e. < 0.1, the actual *P*‐value is shown.

As MAIT cell frequencies in blood have been reported to decline with old age,^29^ we focussed the analysis of MAIT cell frequencies in the group of sepsis patients below the age of 65 (*n* = 29 patients; age range 30–65 years, mean 55, median 59), which was sex and age matched to our healthy donor group collected before or at the beginning of the COVID‐19 pandemic (*n* = 10 patients, age range 40–65 years, mean 52, median 51). We initially performed unsupervised analysis of CD3^+^ single live cell events in the whole flow cytometry data set of the younger sepsis patients and healthy donors using Uniform Manifold Approximation and Projection (UMAP). Projections of the defining markers on the UMAP topography allowed visualisation of the distinct T‐cell subsets, which were confirmed by manual gating (Figure 2a and b). Projection of the sepsis patients and healthy donors separately revealed a marked loss of the topography depicting MAIT cells in sepsis patients (Figure 2c). The decline in MAIT cell frequency was further confirmed by manual gating (*P* = 0.041) (Figure 2d), whereas no significant decreases in CD4^+^, CD8^+^ or double negative (DN) non‐MAIT T cells were observed (Figure 2d). In addition, the frequency of a Vα7.2^+^ CD4^+^ CD161^−^ cluster was reduced in sepsis compared with healthy donors (Figure 2c and d). Analysis of longitudinal paired samples from five sepsis patients indicated persistently low, or even declining, frequency of MAIT cells at Days 2 and 5 after presentation at the emergency department, although this trend did not reach statistical significance (Figure 2e).

We next tested whether the presence of bacteria in the blood was associated with changes in T‐cell subsets among sepsis patients. Indeed, MAIT cell frequencies were reduced in sepsis patients with positive blood cultures compared to those without detectable bacteremia (Figure 2f). MAIT cells in patients with positive blood cultures also expressed higher levels of HLA‐DR (Supplementary figure 2a). Levels of IL‐15 were significantly higher in patients with positive blood cultures, but there were no significant differences for IL‐12 (*P* = 0.09) and IL‐18 (Supplementary figure 2b). The frequency of DN MAIT cells correlated negatively, while CD25^+^ MAIT cells correlated positively with the time to positive blood culture (Supplementary figure 2c). The frequencies of conventional CD8 or CD4 T cells or DN non‐MAIT T cells were similar between sepsis patients with positive and negative blood cultures. Furthermore, there was no difference in MAIT cell percentage between patients with viral sepsis compared with non‐viral sepsis (Figure 2g). Sepsis patients with *S. aureus* in the blood trended towards lower levels of MAIT cells compared to those with *S. pneumoniae* (*P* = 0.055) (Supplementary figure 2d). As MAIT cells have been shown to be particularly susceptible to lysis by the *S. aureus* pore‐forming toxin LukED (18), whole genome sequencing was performed on four of the *S. aureus* isolates. The *luk*ED gene was identified in three of these (Supplementary table 3). Taken together, these results indicate that the previously reported decline in MAIT cells in sepsis patients occurs already in the emergency department and is most evident in bacteremic sepsis.

### Early MAIT cell activation profile separates sepsis and non‐sepsis patients

**Figure 3:**
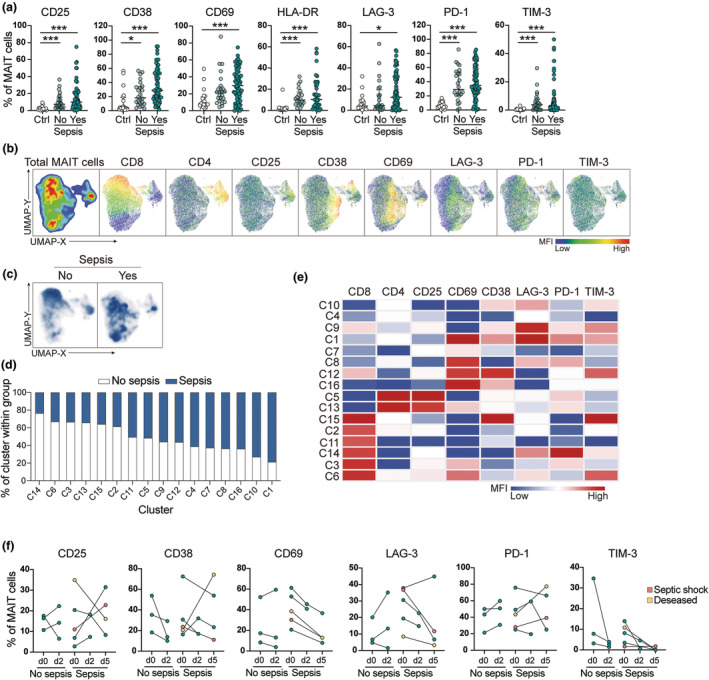
MAIT cells are activated early in sepsis. **(a)** Frequency of indicated markers on MAIT cells identified by manual gating in healthy donors and geriatric controls combined (Ctrl), no sepsis and sepsis patients. Data are presented as median ± IQR, and each dot represents an individual patient. Statistical analysis was performed using the nonparametric Kruskal‐Wallis test followed by Dunn's multiple comparisons test. ***P < 0.001, **P < 0.01, *P < 0.05. **(b)** UMAP of all MAIT cells from all patients included in the automated analysis, showing representative expression of phenotypic markers used for clustering. **(c)** UMAP of all MAIT cells from patients used for the analysis, split according to their sepsis status. **(d)** Percentage of 16 PhenoGraph clusters within total cells in sepsis and no sepsis patient groups. **(e)** Expression of phenotypic markers across 16 detected PhenoGraph clusters (column *z*‐score of median expression values). **(f)** Changes over time in frequencies of MAIT cells expressing indicated markers in non‐sepsis and sepsis patients. Each line represents one patient. Only statistically significant differences are indicated by ***P < 0.001, *P < 0.05.

We first determined the MAIT cell activation profile in sepsis patients, non‐sepsis patients and non‐infected controls (healthy donors and geriatric controls combined). Manual gating revealed elevated expression of all measured activation markers in both sepsis and non‐sepsis patients compared with controls. Sepsis patients tended to have a slightly higher frequency of CD38^+^, CD69^+^ and lymphocyte activation gene 3 (LAG‐3)^+^ MAIT cells compared with non‐sepsis patients, although not significantly so (Figure 3a). Moreover, multivariate regression analyses highlighted the above‐mentioned markers, as well as the DN MAIT population. However, it should be noted that the mean accuracy of the regression models was suboptimal (Supplementary figure 3a). These results indicate that single markers are not sufficient to discriminate between sepsis and non‐sepsis patients. Therefore, we employed unsupervised high‐dimensional profiling of the MAIT cell activation markers in sepsis and non‐sepsis patients using UMAP (Figure 3b and c). This revealed distinct topological patterns between sepsis and non‐sepsis patients, where non‐sepsis patients tended to localise to the CD8 high area (Figure 3b and c). To further identify MAIT cell subpopulations and quantify their abundance in sepsis and non‐sepsis patients, we performed PhenoGraph analysis. Among the 16 clusters identified (Supplementary figure 4), four were equally shared between the two patient groups, while 12 showed an increased abundance in either sepsis or non‐sepsis patients (Figure 3d). C1 and C10 were enriched (above 70%) among sepsis patients, whereas cluster C14 dominated among non‐sepsis patients (Figure 3d). CD8 levels were low in clusters C1 and C10, while high in C14 (Figure 3e). LAG‐3 and PD‐1 were elevated in clusters enriched in both septic and non‐sepsis patients. CD69 was highly expressed in the sepsis‐associated C1 cluster but not in C10. Longitudinal samples (Days 0, 2 and 5) were available from a few of the patients, allowing for assessment of the individual MAIT cell activation markers over time. The levels of CD69 and T‐cell immunoglobulin and mucin‐3 (TIM‐3) decreased with time, but the other markers remained high (Figure 3f). Taken together, the results show that MAIT cells are activated in both sepsis and non‐sepsis patients but differ in their activation profiles.

### MAIT cell activation profiles correlate with inflammatory cytokines and lymphopenia

**Figure 4:**
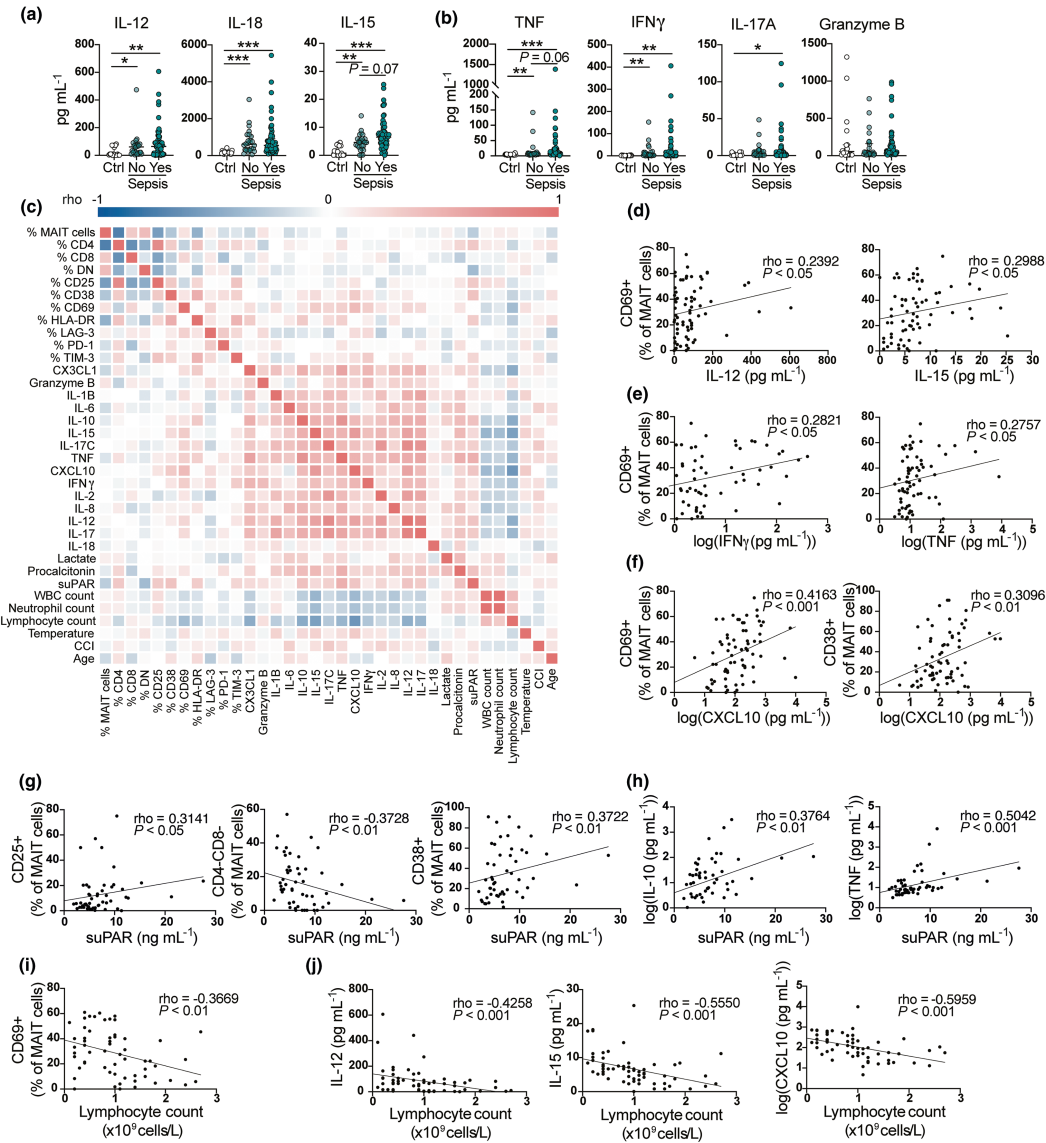
MAIT cell activation in sepsis patients correlates with inflammatory cytokine levels and lymphopenia **(a, b)**. Concentration of **(a)** IL‐12, IL‐18 and IL‐15 and **(b)** TNF, IFNγ, IL‐17A and granzyme B in plasma of healthy donors and geriatric controls combined (Ctrl), non‐sepsis and sepsis patients. Data are presented as median ± IQR, and each dot represents an individual patient. Statistical analysis is performed using the nonparametric Kruskal‐Wallis test, followed by Dunn's multiple comparisons test. Statistically significant differences are indicated by ***P < 0.001, **P < 0.01, *P < 0.05. In case of borderline significant, i.e. < 0.1, the actual *P*‐value is shown. **(c)** A heatmap displaying pairwise Spearman correlations between the frequency of surface markers on MAIT cells, soluble factors in plasma, and clinical parameters in sepsis patients. Colour indicates the strength of the correlation. *P* and rho values are shown in Supplementary table 4. **(d)** Spearman correlations between CD69 expression on MAIT cells and IL‐12 in plasma and CD69 expression and IL‐15 in plasma in sepsis. **(e)** Spearman correlations between CD69 expression on MAIT cells and IFNγ (log_10_‐transformed) in plasma and CD69 expression and TNF (log_10_‐transformed) in plasma in sepsis. **(f)** Spearman correlations between CD69 expression on MAIT cells and CXCL10 (log_10−_transformed) in plasma and CD38 expression and CXCL10 (log_10_‐transformed) in plasma in sepsis. **(g)** Spearman correlations between CD25 and CD38 expression on MAIT cells and suPAR in plasma and CD4^−^CD8^−^ MAIT cells and suPAR in plasma in sepsis. **(h)** Spearman correlations between IL‐10 (log_10_‐transformed) and suPAR and TNF (log_10_‐transformed) and suPAR in plasma in sepsis. **(i)** Spearman correlations between CD69 expression on MAIT cells and lymphocyte counts. **(j)** Spearman correlations between IL‐15, IL‐12, or CXCL10 (log_10_‐transformed) in plasma and lymphocyte counts. The Spearman correlation coefficient (rho) and the associated calculated *P*‐value (p) are indicated on graphs. ***P < 0.001, **P < 0.01, *P < 0.05.

As MAIT cells are major producers of cytokines in TSS (24, 25), we next assessed whether MAIT cell activation could be linked to the levels of inflammatory markers in the plasma. The MAIT cell‐activating cytokines IL‐12, IL‐15 and IL‐18, as well as TNF, IFNγ and IL‐17A, cytokines known to be produced by MAIT cells, were elevated in the plasma of sepsis patients as well as non‐sepsis patients, compared with non‐infected controls (Figure 4a and b). The multivariate regression analysis showed that IL‐15 and IL‐18 contributed to the differentiation between sepsis and non‐sepsis patients (Supplementary figure 3b). Correlation analyses of MAIT cell activation markers and soluble factors in sepsis patients revealed positive, albeit weak, correlations between the MAIT cell CD69 expression and the levels of IL‐12 and IL‐15 and with IFNγ and TNF in the plasma (Figure 4c–e, Supplementary table 4). There was also a positive correlation between the CD69 and CD38 expression levels and plasma levels of the chemokine CXCL10 (Figure 4c and f). Soluble Urokinase Plasminogen Activator Receptor (suPAR), an inflammatory marker of poor prognosis,^30^, ^31^ correlated positively with CD25 expression on MAIT cells and negatively with CD4^−^CD8^−^ MAIT cells (Figure 4c and g). suPAR also correlated positively with IL‐10 and TNF (Figure 4c and h).

We also investigated whether MAIT cell activation profiles correlated with clinical parameters in sepsis patients (Figure 4c, Supplementary table 4). CD69 expression on MAIT cells correlated negatively with the lymphocyte count in the blood (Figure 4c and i). The majority of the soluble factors also correlated negatively with the lymphocyte count, in particular the MAIT cell‐activating cytokines IL‐12 and IL‐15 and the MAIT cell‐attracting chemokine CXCL10 (Figure 4c and j). All soluble factors measured, apart from granzyme B, correlated positively with procalcitonin levels (Figure 4c, Supplementary table 4). Taken together, these results indicate that MAIT cell activation is associated with an elevated systemic inflammatory response as well as lymphopenia.

### MAIT cell phenotypes are associated with disease outcomes, organ dysfunction and clinical endotypes

**Figure 5:**
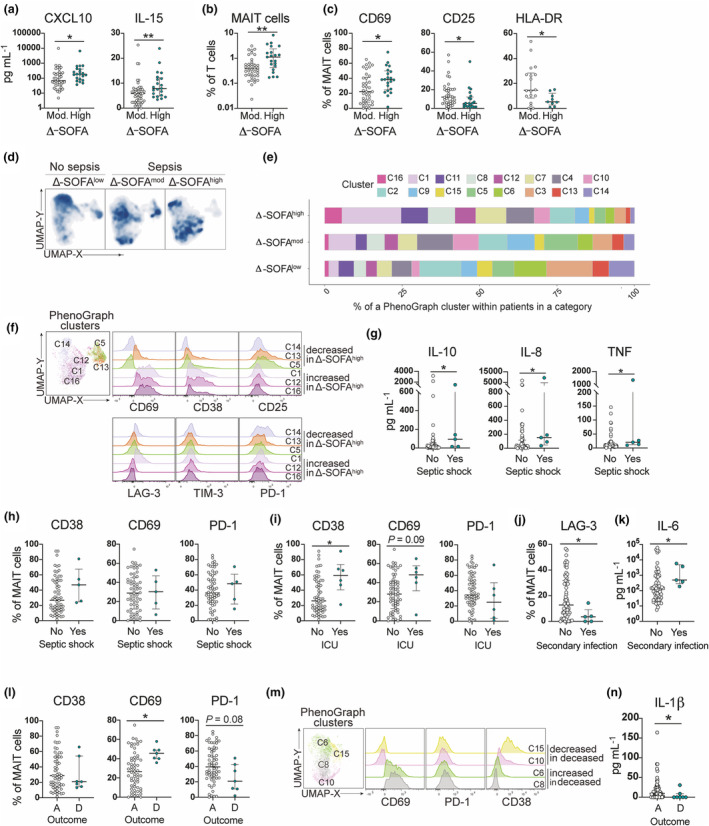
MAIT cell activation profiles associated with clinical parameters and outcomes in sepsis patients. **(a)** Concentration of CXCL10 and IL‐15 in plasma, **(b)** MAIT cell frequencies among total CD3^+^ cells and **(c)** frequencies of CD69^+^, CD25^+^ and HLA‐DR^+^ MAIT cells in sepsis patients with moderate (2, 3) or high (≥ 4) Δ‐SOFA score. **(d)** UMAP of all MAIT cells from patients with or without sepsis, split according to their Δ‐SOFA score. **(e)** Percentage of 16 PhenoGraph clusters within total cells in Δ‐SOFA^high^, Δ‐SOFA^mod^ and Δ‐SOFA^low^ patient groups. **(f)** Expression of indicated markers in selected PhenoGraph clusters that are over‐ or under‐represented in Δ‐SOFA^high^ patient group. **(g)** Concentrations of IL‐10, IL‐8 and TNF in sepsis patients with or without septic shock. **(h, i)** Frequencies of CD38^+^, CD69^+^ and PD‐1^+^ MAIT cells in sepsis patients **(h)** with or without septic shock and **(i)** patient treated in ICU or not. **(j)** Frequencies of LAG‐3^+^ MAIT cells in sepsis patients who developed secondary infections within 60 days. **(k)** Concentration of IL‐6 in plasma of sepsis patients who developed secondary infections within 60 days. **(l)** Frequencies of CD38^+^, CD69^+^ and PD‐1^+^ MAIT cells in sepsis patients who were alive, A, or died, D, within 28 days after the onset of sepsis. **(m)** Expression of indicated markers in selected PhenoGraph clusters that are over‐ or under‐represented in sepsis patients who died within 28 days after the onset of sepsis. **(n)** Concentration of IL‐1β in plasma of sepsis patients who were alive, A, or died, D, within 28 days after the onset of sepsis. **(a–c, g– l, n)** Data are presented as median ± IQR, each dot representing an individual patient. Statistical analysis was performed using the nonparametric Mann–Whitney test. Statistically significant differences are indicated by **P < 0.01, *P < 0.05. In case of borderline significance, i.e. < 0.1, the actual *P*‐value is shown.

Next, we assessed the MAIT cell activation in relation to disease severity and outcome. The SOFA score was used as a sepsis severity measure. As per the sepsis definition, all the non‐septic patients had a Δ‐SOFA score below 2 (low). The sepsis patients were divided into moderate (2, 3) and high (≥ 4) Δ‐SOFA score. The plasma levels of IL‐15 and CXCL10 were elevated in patients with high Δ‐SOFA score compared with moderate (Figure 5a). Patients with high Δ‐SOFA had higher MAIT cell frequencies than patients with a moderate Δ‐SOFA score (Figure 5b). Manual gating revealed that patients with high Δ‐SOFA score had higher expression of CD69 but lower expression of CD25 and HLA‐DR on MAIT cells than patients with a moderate Δ‐SOFA score (Figure 5c). Similarly, the patients with high Δ‐SOFA localised to the CD69 high region of the UMAP topography (Figures 5d and 3b). PhenoGraph analysis showed an enrichment of clusters C1, C12 and C16 in Δ‐SOFA high patients and decreased abundance of C14, C13 and C5 (Figure 5e and f). Clusters with increased representation in Δ‐SOFA high individuals were characterised by high CD69 and CD38 expressions, while clusters with decreased representation in Δ‐SOFA high patients expressed CD25 (Figure 5f). There was no difference in any of the tested markers in non‐MAIT T cells (Supplementary figure 5a).

Among the sepsis patients, we also assessed the MAIT activation profile in relation to the development of septic shock, the need for ICU care, the development of secondary infections and the outcome. Although levels of IL‐8, IL‐10 and TNF were elevated in the plasma of patients with septic shock (Figure 5g), there was no difference in MAIT cell activation marker expression (Figure 5h). In patients requiring ICU care, CD38 expression was higher on MAIT cells (Figure 5i), but not on non‐MAIT T cells (Supplementary figure 5b). Patients who developed secondary infections had lower LAG‐3 expression on MAIT cells (Figure 5j) and higher levels of IL‐6 in plasma (Figure 5k). The seven patients who died within 28 days after the onset of sepsis expressed significantly elevated levels of CD69 on MAIT cells, while slightly lower levels of PD‐1 (Figure 5l). In contrast, there was no difference in CD69 expression on non‐MAIT T cells in patients who died compared with survivors, whereas non‐MAIT T cells in patients who died expressed significantly lower levels of PD‐1 (Supplementary figure 5b). The association between CD69 expression and a fatal outcome was also evident in the PhenoGraph analysis, where the clusters enriched in deceased patients (C1, C6 and C8) were high in CD69 (Figure 5m). Plasma levels of IL‐1β were lower in the patients who died than in the survivors (Figure 5n).

**Figure 6:**
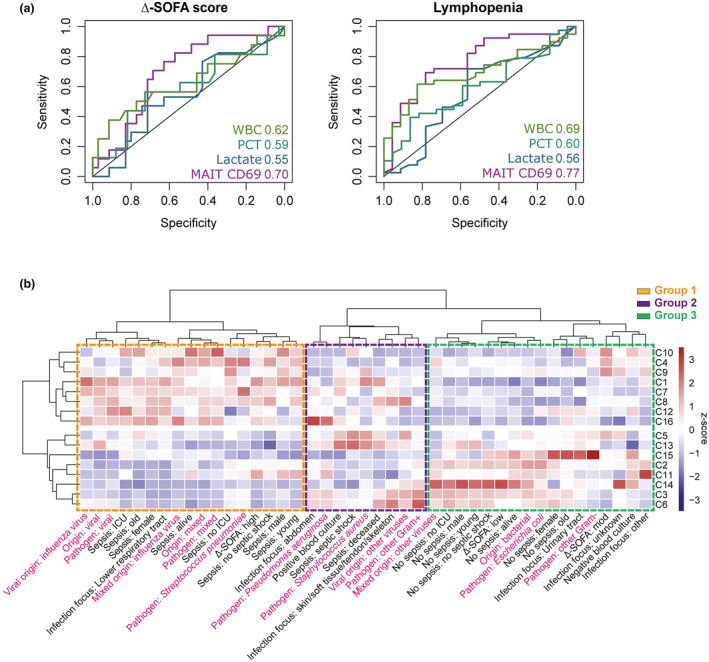
The MAIT cell phenotype in sepsis is associated with clinical endotypes. **(a)** Receiver operating characteristic (ROC) analyses for high versus moderate Δ‐SOFA and for lymphopenia. The ROC plots show results, including the AUC values, of MAIT CD69 expression and the clinical markers white blood cells (WBC), procalcitonin (PCT) and lactate. **(b)** Hierarchical clustering of PhenoGraph clusters and clinical categorical parameters. The heatmap was calculated as column z‐score of cluster percentages within each clinical categorical parameter group. Putative MAIT cell endotypes are indicated as group 1 (orange), group 2 (purple) and group 3 (green).

To further test the predictive value of MAIT CD69 expression, ROC analyses were done for high versus moderate Δ‐SOFA as well as for lymphopenia. The analyses yielded an AUC of 0.70 (specificity 0.69; sensitivity 0.71) for Δ‐SOFA and an AUC of 0.77 (specificity 0.78; sensitivity 0.69) for lymphopenia. Notably, MAIT CD69 expression outperformed selected clinical markers including WBC, procalcitonin and lactate (Figure 6a, Supplementary table 5).

To further explore the MAIT cell phenotype in relation to clinical parameters, we performed a comprehensive hierarchical clustering of the abundance of PhenoGraph clusters across clinical categorical parameters, including microbiological data, infection focus, sepsis or not, ICU treatment, septic shock, mortality, Δ‐SOFA, as well as age and sex (Figure 6b). This revealed distinct separation of specific sepsis clinical endotypes into three main groups, including: Group 1, patients with lower respiratory tract infections, high Δ‐SOFA and pneumococcal or viral infections; Group 2, patients with bacteremia, septic shock, fatal outcome, *S. aureus* or other Gram‐positive bacterial infections; and Group 3, patients with no sepsis, low or moderate Δ‐SOFA, urinary tract infections, *E. coli* or other Gram‐negative bacteria (Figure 6b). Notably, patients with a fatal outcome and high Δ‐SOFA were found in different groups. As previously noted, they shared the C1 cluster characterised by CD69^+^ MAIT cells but showed marked differences in the other clusters. Among patients with less severe disease found in Group 3, cluster C14 was predominant. Notably, C14 had the highest PD‐1 expression (Figure 3e), in line with the association between high PD‐1 and survival (Figure 5l). These data show that the early MAIT cell activation status can be linked to clinical parameters, disease severity and outcome. In particular, elevated expression of CD69 on MAIT cells is associated with organ dysfunction and mortality.

## Discussion

Sepsis is a rapidly progressing syndrome, with early detection and intervention being critical for patient survival. However, diagnosis is often challenging because of sometimes vague symptoms and a heterogeneity in this patient group. An important advancement for sepsis identification is the implementation of a clinical sepsis alert system at the emergency department.^32^, ^33^ In this study, we utilise a patient cohort with suspected sepsis identified through this system to seek early markers of value for diagnosis and prognostication already at the admittance to the emergency department. Focussing on MAIT cells as rapid responders to infections, we identify MAIT cell phenotypes discriminating between sepsis and non‐sepsis patients, as well as phenotypes linked to specific sepsis endotypes and outcomes, indicating their potential as early diagnostic and prognostic biomarkers.

MAIT cell frequencies declined in sepsis patients compared with healthy controls, while the frequencies of CD4^+^, CD8^+^ or DN non‐MAIT T cells remained unchanged. This had previously been reported in sepsis patients in the ICU,^23^, ^24^, ^25^, ^26^, ^27^, ^28^ and here, we extend these findings and show reduced MAIT cell frequencies in circulation already at the time of presentation at the emergency department. Although there was no significant decline in total CD4^+^ non‐MAIT T cells, we found that the frequency of Vα7.2^+^ CD4^+^ CD161^−^ T cells was reduced in sepsis patients compared with healthy donors. Further delineation of this population was not possible with the flow cytometry panel used here, but both the identity and the potential role of these cells in sepsis would be interesting to investigate further. The loss of MAIT cells in circulation could be because of migration to tissues, which is supported by studies in mice,^34^, ^35^ but could also be because of activation‐induced cell death.^36^ Furthermore, bacteremic patients had a higher degree of MAIT cell loss as well as higher plasma levels of inflammatory cytokines. Whether this reflects an impaired infection control because of low frequency of MAIT cells or that the bacteremia‐elicited systemic activation results in loss of MAIT cells remains to be elucidated.

Despite the overall reduced MAIT cell frequency in sepsis patients, higher MAIT cell frequencies were associated with increased organ dysfunction, as defined by the Δ‐SOFA score. Possibly, this could be because of increased proliferation of MAIT cells or failure of these cells to migrate into the tissues in the more severely ill sepsis patients in the beginning of sepsis progression. Our data show that the MAIT cell activation phenotype was associated with clinical parameters, poor outcome and disease severity. In particular, we identified distinct MAIT cell clusters associated with either sepsis (C1) or non‐sepsis patient groups (C14). The CD69 high C1 cluster with highest enrichment in sepsis patients was also dominant in fatal cases, septic shock and in patients with a high Δ‐SOFA score. The MAIT cell activation profile may therefore potentially be used to discriminate high‐risk patient subgroups. CD69 is a promising marker for this purpose. Manual gating confirmed that high CD69 expression on MAIT cells is associated with a fatal outcome, organ dysfunction (assessed by the Δ‐SOFA score) and lymphopenia. A similar association between CD69^+^ MAIT cells and poor outcome has been described in critically ill patients including ICU sepsis and COVID‐19.^27^, ^37^, ^38^ In contrast, Choi *et al*.^28^ found a decrease in CD69 expression on MAIT cells in ICU sepsis patients. As we found that the elevated CD69 levels at the time of admission to the emergency department declined at Days 2 and 5, the timing of CD69 measurement is likely critical, and our findings indicate that CD69 is particularly useful as an early prognostic marker.

The microbiological aetiology is one important factor contributing to the heterogeneous host response associated with sepsis.^5^ MAIT cells respond to microbial riboflavin metabolite antigens,^11^ bacterial superantigens,^21^, ^22^ and innate cytokines^15^ produced in response to both viruses and bacteria and display a microbe‐specific heterogeneous response pattern.^14^ Hierarchical clustering analysis of early MAIT cell activation profiles revealed separation of clinical sepsis endotypes. Notably, there was a clear separation between patients with the two most common sepsis foci, where lower respiratory tract infection clustered with high Δ‐SOFA, *S. pneumoniae* and viral infection, while urinary tract infection clustered with less severe sepsis or non‐sepsis, as well as *E. coli*.

In this study, it is not possible to conclude whether the MAIT cell activation contributes to sepsis severity or is an indicator of other factors underlying poor outcome. MAIT cells have both been suggested to promote disease progression^21^, ^22^ and to have a protective role in an experimental murine sepsis model.^23^ In the present study, we find that CD69 expression correlates both with cytokines stimulating MAIT cell activation and with cytokines produced by MAIT cells, such as IFNγ and TNF, which may indicate that MAIT cells contribute to the pro‐inflammatory cytokine response in sepsis. This is in line with our previous study identifying MAIT cells as significant contributors to the cytokine storm in STSS.^21^ However, Choi *et al*.^28^ reported that the responsiveness of MAIT cells to microbial stimuli decreased over time in sepsis. Persistent depletion of MAIT cells was reported to be associated with increased incidence of ICU‐acquired infections,^24^ also pointing to the importance of a functional MAIT cell compartment in later stages of sepsis and secondary infections. Among the five patients in our cohort who developed secondary infections, MAIT cells displayed lower LAG‐3 expression. Whether this is linked to a dysfunctional response increasing susceptibility to subsequent infections remains to be elucidated. Therefore, it remains to be further delineated whether MAIT cells have a damaging or protective effect in various stages of sepsis.

It is important to note that this study has limitations. First of all, the cohort is limited in size, which impacts the statistical power in specific subgroup analyses, in particular analyses of outcomes related to septic shock, ICU and death. Second, the samples and flow cytometry data were collected over a two‐year period, which may introduce variability in some readouts. We have therefore refrained from comparing the mean fluorescence intensity in the manual gating analysis. Both sepsis alert groups were collected simultaneously, and the younger healthy control group was collected by the end of the sepsis alert collection. Therefore, any potential differences in the flow cytometry measurements would affect the patient groups equally. However, because of the COVID‐19 pandemic, the geriatric control group was collected 2 years after the other samples. Comparison of this group with the patient groups has therefore been done with caution, and these samples have not been included in the unsupervised analysis. A third limitation is that we collected samples at one time point only for most patients, and therefore, the kinetics and dynamics of the MAIT cell response during sepsis progression remain uncertain. It would also have been of value to have access to convalescent samples to investigate whether the MAIT cell compartment normalises with time.

A notable strength of this study is the early time point at which the samples were collected and that samples were collected before the sepsis diagnosis was determined. This allows us to compare the sepsis patients to non‐septic patients who displayed signs of sepsis upon arrival at the hospital. Early detection and treatment are crucial for patient survival and for the identification of early immune alterations that can be used for prognostication, diagnosis or as therapeutic targets.

In summary, we have found sepsis clinical endotype‐specific MAIT cell phenotypes in patients admitted to the emergency department. Notably, high expression of CD69 was associated with a poor outcome and organ dysfunction. The MAIT cell activation profile may therefore provide candidate early prognostic markers in sepsis.

## Methods

### Study design and patient cohort

Patients with suspected sepsis were recruited at the Karolinska University Hospital Huddinge, Stockholm, Sweden, from October 2018 to March 2020. All patients at the Emergency Department are routinely subjected to triage with the Rapid Emergency Triage and Treatment System.^32^ For patients with high triage scores, indicating organ dysfunction, combined with symptoms of infection (fever, history of fever or clinical suspicion of infection), the emergency department nurse triggers the sepsis alert, as previously described.^39^, ^40^ The triage parameters used are described in detail in Supplementary table 1.

Blood samples were collected in 5‐mL EDTA tubes at presentation in the emergency department in patients who triggered the sepsis alert. PBMC were isolated and stained fresh for flow cytometry, and plasma was stored at −80°C. Bacterial cultures, measures of blood lactate and serum procalcitonin levels, and counting of white blood cells, neutrophils and lymphocytes were performed at the emergency department. After sample collection, the sepsis alert patients were divided into groups with sepsis (*n* = 69), infection but no sepsis (*n* = 26) and no infection (*n* = 9; excluded from the study). Based on the Sepsis‐3 criteria,^1^ sepsis was defined as infection and a change in sequential organ failure assessment (Δ‐SOFA) score ≥ 2. The Δ‐SOFA score was calculated using the SOFA at admission to the emergency department minus the baseline SOFA. For baseline SOFA, the last available parameters from outpatient clinics in Region Stockholm in the time window 7–90 days prior to admission to the emergency department were used. When SOFA parameters were missing within this baseline time window, the baseline SOFA was assumed to be 0. The sepsis group was further divided into subgroups based on the development of septic shock (according to the Sepsis‐3 criteria^1^), 28‐day survival, treatment at the ICU, positive blood cultures (bacteremia), viral infection, immunosuppressive therapy, infection focus and development of secondary infection within 60 days. The presence of infection on admission was assessed by an Infectious Diseases specialist (ÅP and/or KS), based on the clinical picture, radiological results, laboratory results and microbiological results. A detailed description of infection focuses is found in Supplementary table 1. The presence of secondary infection within 60 days from admission was assessed by an Infectious Diseases specialist (HA and/or KS), based on the criteria for infections in critically ill from the Centers for Diseases Control and Prevention.^41^

Longitudinal sampling with repeated EDTA blood samples on Days 2 and 5 after presentation at the emergency room was performed in eight study patients.

Blood samples were also taken from 10 healthy donors (40–65 years old) recruited at the blood bank at the end of the sepsis recruitment period. None of the healthy donors were infected with SARS‐CoV‐2 at the time of sampling. Because of the COVID‐19 pandemic, it was difficult to recruit older healthy donors, but during spring 2022, after the COVID‐19‐related restrictions were lifted, a second control group (*n* = 10, 76–98 years old) was recruited at a geriatric ward at Karolinska University Hospital Huddinge. Inclusion and exclusion criteria for the geriatric control patients are described in Supplementary table 1.

### PBMC isolation and staining

PBMC were isolated from freshly collected peripheral blood by Ficoll–Hypaque density gradient centrifugation (Lymphoprep, Axis‐Shield, Dundee, United Kingdom). Cells were washed twice in PBS with 2% FCS and 4 mM EDTA and stained for cell surface markers in a 96‐well plate for 20 min, followed by fixation in CellFix (BD Biosciences, Franklin Lakes, New Jersey, USA) for 15 min. The antibodies used are listed in Supplementary table 6. All stainings were performed at 4°C.

### Flow cytometry

Samples were acquired on an LSR Fortessa flow cytometer (BD Biosciences) equipped with 355‐, 405‐, 488‐, 561‐ and 639‐nm lasers. FCS3.0 files were exported from the BD FACSDiva software and imported into the FlowJo software v. 10. Automated compensation matrixes were generated using polystyrene beads (BD Biosciences), and the compensation platform in the FlowJo software. The compensated data set was used both for downstream manual gating and for automated analysis. The gating strategy is shown in Supplementary figure 1. For the unsupervised analysis, events were first down‐sampled from the CD3^+^ gate or MAIT cell gate across all samples using the DownSample plugin. FCS 3.0 files of CD3^+^ cells or MAIT cells from each patient with the corresponding compensation matrices applied were imported into FlowJo v10.8.2 for the high‐dimensional data analysis. The following FlowJo plugins were used: UMAP (v3.1) and PhenoGraph (v3.0). Clinical data were added to each patient sample as categorical parameter keywords and concatenated for analysis. UMAP was run on the CD3^+^ events including CD4, CD8, Vα7.2, CD161, FSC and SSC measurements and on Vα7.2^+^ CD161^+^ cells using CD8, CD4, CD25, CD38, CD69, LAG‐3, PD‐1 and TIM‐3. PhenoGraph was run on Vα7.2^+^ CD161^+^ cells using the same parameters, with *k* = 30. Because of uneven number of patients represented in each clinical parameter group (i.e. over‐ and under‐represented input groups were similarly weighted in the PhenoGraph output clusters), we normalised the PhenoGraph output clusters to account for the total number of cells from each input group. Significant PhenoGraph clusters (*P* ≤ 0.05) were determined by chi‐squared goodness‐of‐fit tests comparing the relative abundance of each categorical group in each individual PhenoGraph cluster relative to input. Where indicated, *z*‐score of median fluorescence intensity (MFI) was calculated as follows: *Z* = $z=\frac{\left(x-\mu \right)}{\sigma }$, with *x* = raw score, *μ* = mean of sample distribution and *σ* = standard deviation. Figures were generated in R (v4.2.2) and RStudio (v2022.07.2) with packages tidyr (v1.2.1), dplyr (v1.0.10), ggplot2 (v3.4.0), pheatmap (v1.0.12) and randomcoloR (v1.1.0.1).

### Multiplex assay of plasma inflammatory mediators

Plasma was collected from each blood sample by spinning 500 μL of blood at 1000 *g* for 5 min. The plasma was stored at −80°C until further analysis. Before the assay, the plasma was centrifuged at 16000 *g* for 4 min and diluted 1:2 and 1:100. CX3CL1, granzyme B, IL‐1β, IL‐6, IL‐10, IL‐15, IL‐17C, IL‐17A, TNF, CXCL10, IFNγ, IL‐2, IL‐12, IL‐8 and IL‐18 levels were determined using a customised 15‐plex Luminex Assay (R&D Systems, Minneapolis, Minnesota, USA) and analysed on the Bio‐Plex MAGPIX reader (Bio‐Rad, Hercules, California, USA).

### ELISA

suPAR concentration in plasma was determined using the suPARnostic® ELISA Kit (ViroGates, Birkerød, Denmark) according to the manufacturer's instructions.

### Whole genome sequencing

Bacteria were pretreated by lysozyme and lysostaphin, followed by the extraction of genomic DNA with the EZ1 Advanced XL system (QIAGEN, Hilden, Germany), as previously described.^42^ Sequencing was performed on the Illumina platform, generating paired‐end (2 × 150 bp) sequences in ≥ 100× coverage (SciLifeLab, Stockholm, Sweden). Multi‐locus sequence typing (MLST) was determined from whole genome sequences using the 1928 Diagnostics online platform (1928 Diagnostics, Gothenburg, Sweden). Virulence factors were identified by using VirulenceFinder 2.0 (Center for Genomic Epidemiology, Denmark).

### Statistics

Statistical analyses were performed using Prism V8.4.3. Nonparametric Mann–Whitney tests were used to determine statistically significant differences between two unpaired groups, and the Kruskal–Wallis test, followed by Dunn's multiple comparisons test, was used when comparing more than two unpaired groups. Two parameter correlations were calculated using the Spearman correlation. *P < 0.05, **P < 0.01, ***P < 0.001. The multivariate regression analyses were performed on R (v.4.2.3), in R Studio Server (v.2021.09.2.382). Least absolute shrinkage and selection operator (LASSO) method (R package glmnet, v4.1–2) was used to select variables of relevance in differentiating patient groups. The data set was partitioned to build the model with 80% of patients, while the validation set consisted of the remaining 20%. We ran 1000 iterations of logistic regression with lasso regularisation models using alpha = 1 and selecting the minimal lambda for the best model in an iteration. The package pROC was used to generate the Receiver Operating Characteristic (ROC) curves. The threshold with the highest sensitivity and specificity was selected using the ‘closest.topleft’ method.^43^

## Author contributions

**Johanna Emgård:** Conceptualization; data curation; formal analysis; investigation; methodology; visualization; writing – original draft. **Iva Filipovic:** Formal analysis; investigation; methodology; visualization; writing – review and editing. **Christian Unge:** Data curation; resources; writing – review and editing. **Laura M Palma Medina:** Formal analysis; methodology; writing – review and editing. **Åsa Parke:** Data curation; resources; writing – review and editing. **Helena Bergsten:** Investigation; writing – review and editing. **Kirsten Moll:** Investigation; writing – review and editing. **Majda Dzidic:** Investigation; writing – review and editing. **Helena Alpkvist:** Data curation; resources; writing – review and editing. **Hong Fang:** Investigation; writing – review and editing. **Volkan Özenci:** Resources; writing – review and editing. **Niklas K Björkström:** Supervision; writing – review and editing. **Mattias Svensson:** Conceptualization; project administration; writing – review and editing. **Johan K Sandberg:** Funding acquisition; supervision; writing – review and editing. **Kristoffer Strålin:** Conceptualization; data curation; funding acquisition; project administration; writing – review and editing. **Anna Norrby‐Teglund:** Conceptualization; funding acquisition; project administration; supervision; writing – original draft.

## Conflict of interest

The authors declare no conflict of interest.

## Study approval

The study was approved by the Regional Ethics Review Board in Stockholm (2017/1358‐31, 2020–05195). Written informed consent was collected from the sepsis alert patients within a month after the sampling. Samples from patients who did not give consent were discarded and data was erased. Healthy controls and geriatric controls provided written informed consent prior to sampling.

## Supporting information


**Supplementary figures** **1‐5**

**Supplementary tables** **1‐6**

## Data Availability

Additional data and experimental details are available in the Supporting information files.
