# Supplementary material for: Distinct MAIT cell phenotypes associated with sepsis clinical outcome in emergency department patients
Source: Clin Transl Immunology. 2025 Mar 4;14(3):e70028. doi: 10.1002/cti2.70028 (PMC11879382; doi:10.1002/cti2.70028)
Supplement: Supplementary file 1 — Supplementary figures 1‐5 [file CTI2-14-e70028-s002.pdf]

Supporting data for

Distinct MAIT cell phenotypes associated with sepsis clinical outcome in emergency department patients

Johanna Emgård[1], Iva Filipovic[1], Christian Unge[2,3], Laura M Palma Medina[1], Åsa Parke[2,4], Helena Bergsten[1], Kirsten Moll[1], Majda Dzidic[1], Helena Alpkvist[2,4], Hong Fang[5,6], Volkan Özenci[5,6], Niklas K Björkström[1], Mattias Svensson[1], Johan K Sandberg[1], Kristoffer Strålin[2,4,*], Anna Norrby-Teglund[1,*]

[1]Center for Infectious Medicine, Department of Medicine Huddinge, Karolinska Institutet, Stockholm, Sweden
[2]Department of Medicine Huddinge, Karolinska Institutet, Stockholm, Sweden
[3]Functional Area of Emergency Medicine, Karolinska University Hospital, Stockholm, Sweden
[4]Department of Infectious Diseases, Karolinska University Hospital, Stockholm, Sweden
[5]Division of Clinical Microbiology, Department of Laboratory Medicine, Stockholm, Sweden
[6]Department of Clinical Microbiology, Karolinska University Hospital, Stockholm, Sweden
[*]Equal contribution

Corresponding authors

Anna Norrby-Teglund
Email: anna.norrby-teglund@ki.se
Johanna Emgård
Email: johanna.emgard@ki.se

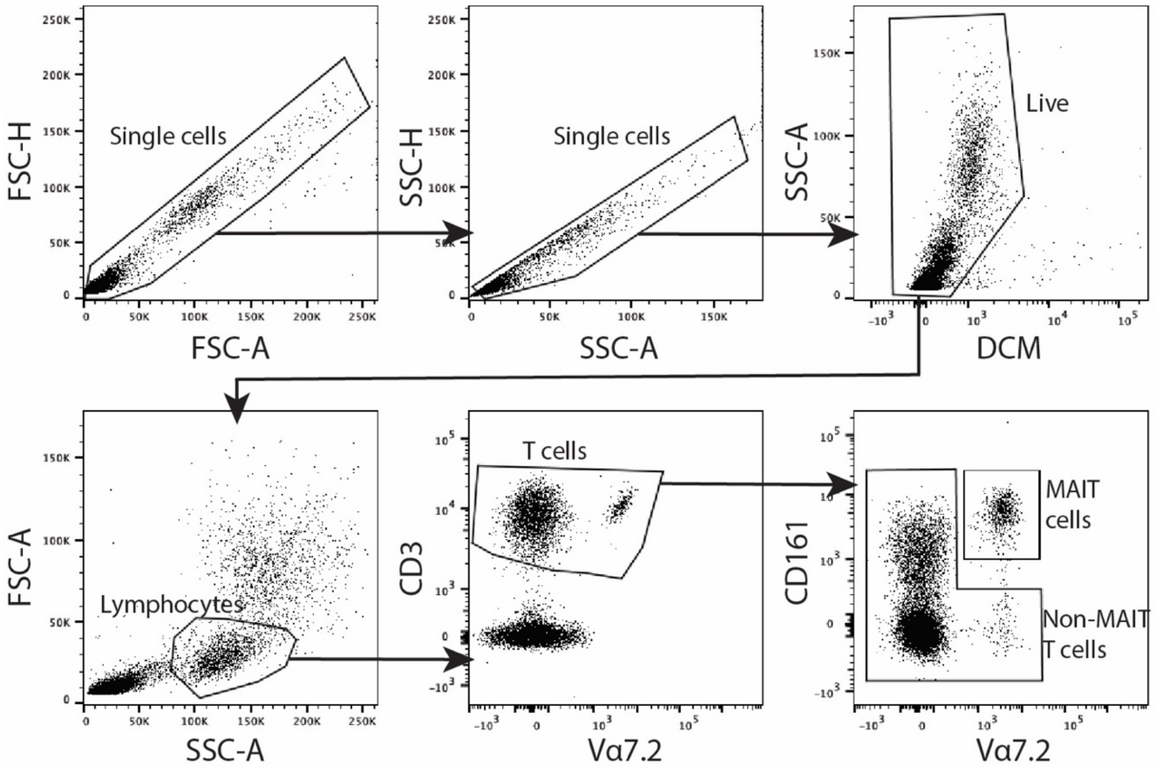

Supplementary figure 1. Gating strategy used for the identification of MAIT cells in PBMC isolated from patients and healthy controls. DCM, dead cell marker.

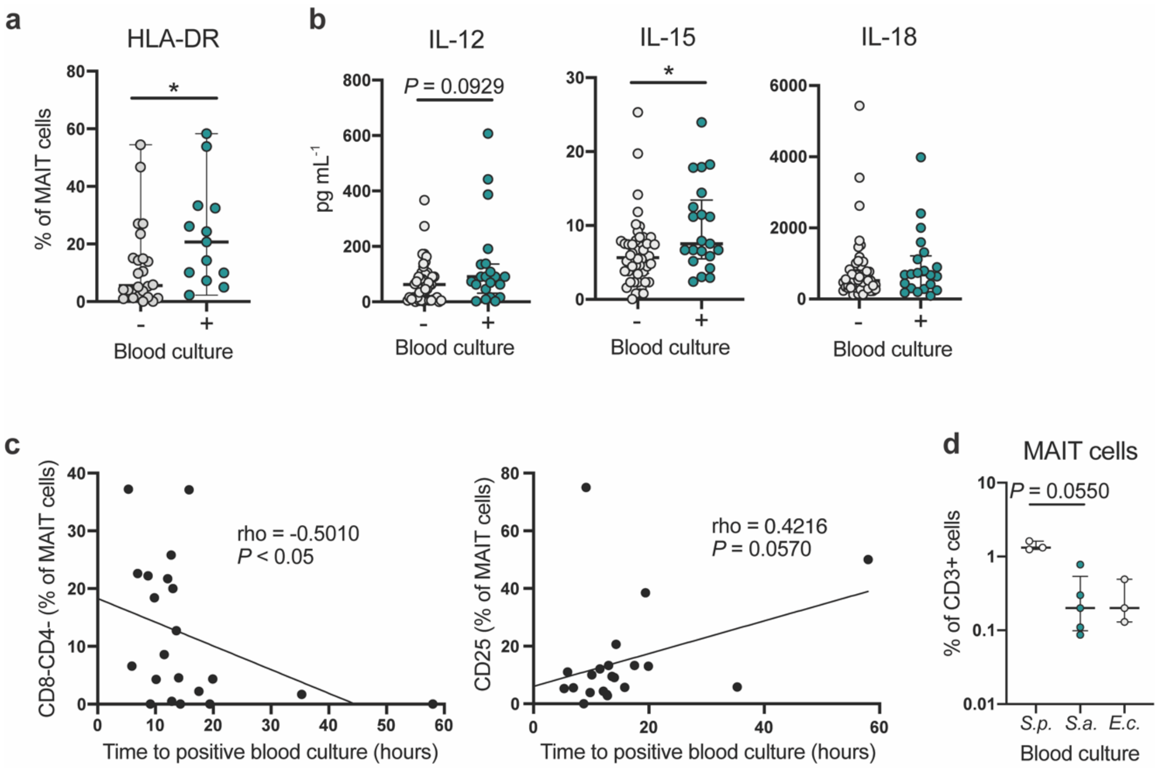

Supplementary figure 2. MAIT cell frequency and activation profiles associated with the bacterial species detected in blood cultures from sepsis patients. (**a**) Expression of HLA-DR on MAIT cells in sepsis patients with negative or positive blood cultures. (**b**) Concentration of indicated soluble factors in plasma of sepsis patients with negative or positive blood cultures. (**c**) Spearman correlations between the frequencies of CD8-CD4- or CD25+ MAIT cells and the time to positive blood culture. (**d**) Frequency of MAIT cells among total CD3+ T cells in sepsis patients with blood cultures positive for *Streptococcus pneumoniae* (*S. p.*), *Staphylococcus aureus* (*S. a.*) or *Escherichia coli* (*E. c.*). Statistical analysis is performed using (**a** and **b**) nonparametric Mann-Whitney test or (**d**) Kruskal-Wallis test followed by Dunn's multiple comparisons test. ****P* <0.001, ***P* <0.01, **P* <0.05. The Spearman correlation coefficient (rho) and the associated calculated *P*-value (p) are indicated on graphs in (**c**).

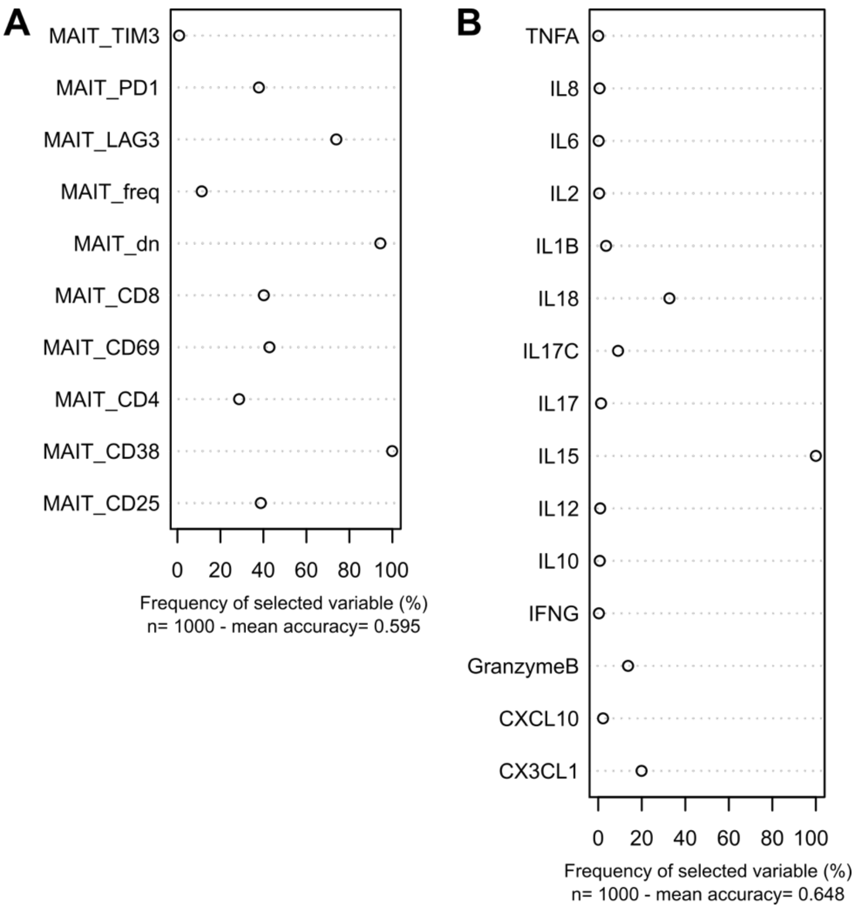

Supplementary figure 3. Multiparameter regression analyses indicate variables that are likely relevant for differentiating cases of sepsis based on (**a**) MAIT cells surface markers or (**b**) soluble factors measured in plasma. The plots show the frequency each variable was selected on the logistic regression models using LASSO regularization (n=1000).

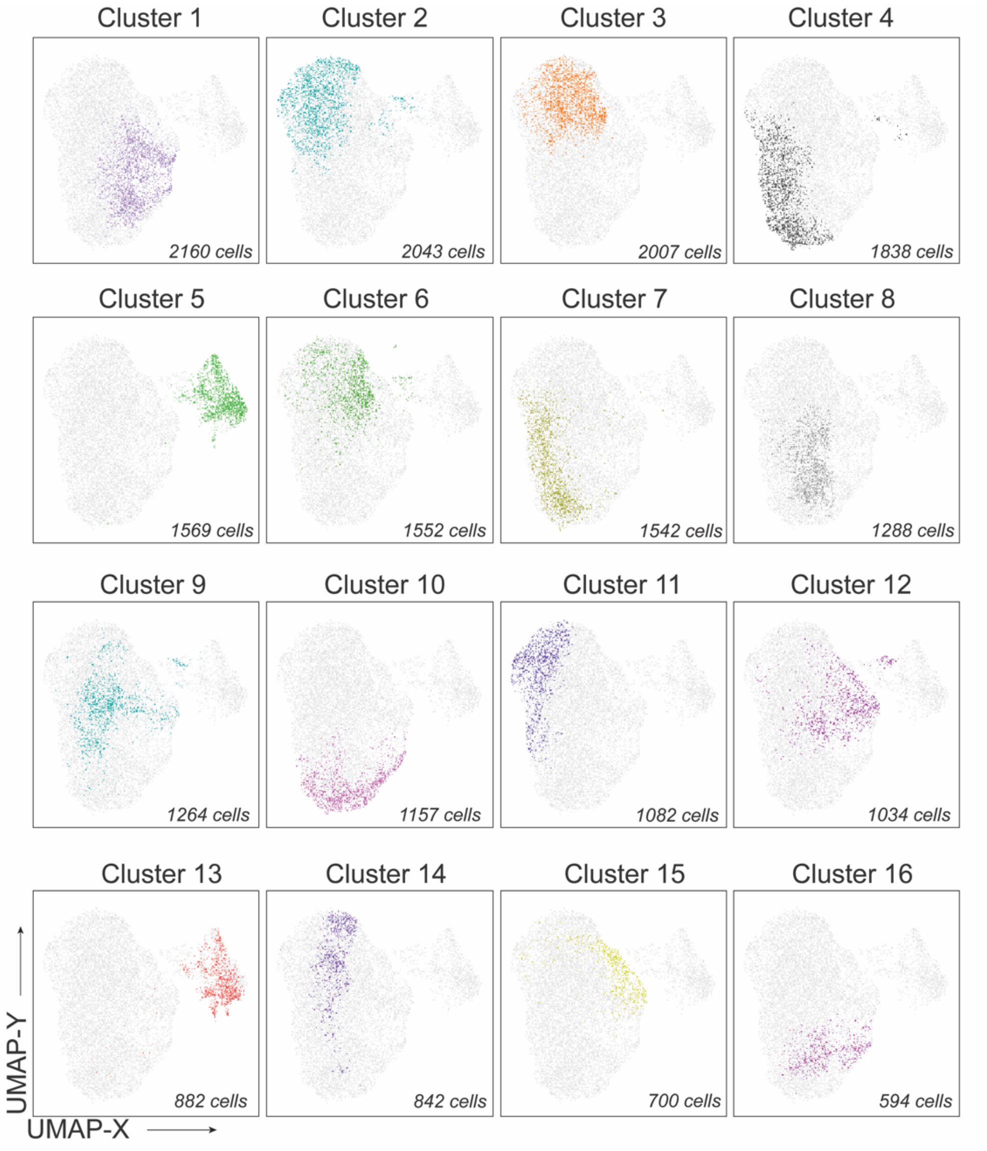

Supplementary figure 4. Overlay of the individual Phenograph clusters over the UMAP projection of MAIT cells from all patients included in the automated analysis. Cell numbers per cluster are indicated in each plot.

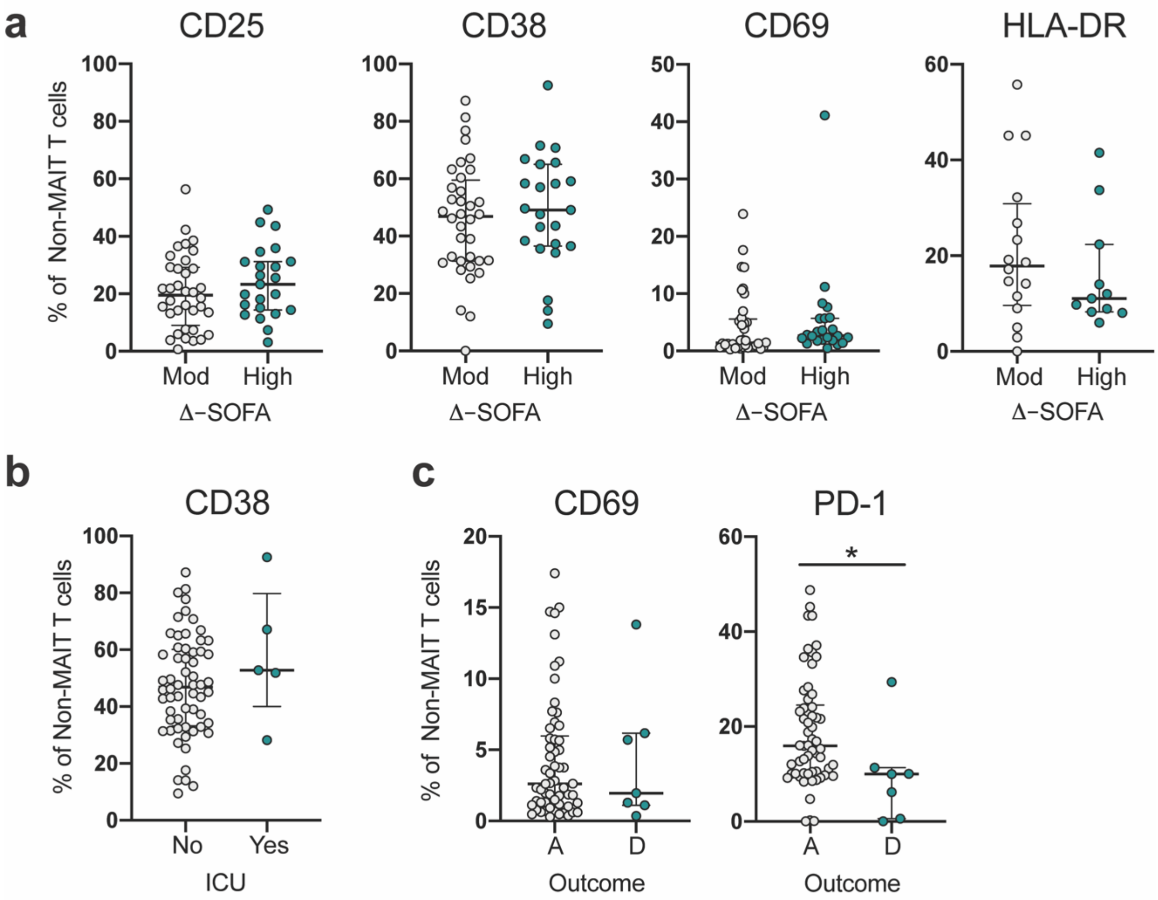

Supplementary figure 5. Non-MAIT T cell activation profiles associated with sepsis severity and outcome. Frequencies of indicated markers on non-MAIT T cells in (**a**) sepsis patients with moderate (2-3) or high (≥4) Δ-SOFA score, (**b**) sepsis patients treated in ICU or not, and (**c**) patients who were alive, A, or died, D, within 28 days after onset of sepsis. Data is presented as median ± IQR, each dot representing an individual patient. Statistical analysis is performed using non-parametric Mann-Whitney test. ****P* <0.001, ***P* <0.01, **P* <0.05.
